# “Sorting Things out”: A Scoping Review of Sexual Homicide Typologies

**DOI:** 10.1002/bsl.2722

**Published:** 2025-03-24

**Authors:** Eric Beauregard, Julien Chopin

**Affiliations:** ^1^ School of Criminology Simon Fraser University Burnaby Canada; ^2^ School of Criminal Justice University of Lausanne Lausanne Switzerland

**Keywords:** classification, scoping review, sexual homicide, typologies

## Abstract

Sexual homicides are complex crimes that have been the focus of numerous classification systems aimed at aiding investigations, understanding offender behavior, and informing treatment plans. Over the past 25 years, a variety of typologies have been developed to categorize these offenses. This scoping review examines these typologies, exploring their evolution and the key offender, victim, and crime characteristics used to define them. The review identifies 19 empirical typologies from Canada, France, the UK, South Africa, and other regions, most of which are based on police and offender data. Typologies typically include categories such as “sadistic” and “anger‐driven” homicides, though the number of types varies across studies. Moreover, the review highlights gaps in current research, such as limited sample sizes and the need for more diverse cultural perspectives. Recommendations are made for developing a more comprehensive and validated typology that incorporates broader data sources and modern methodologies, such as machine learning techniques, to enhance profiling, investigation, and prevention efforts.

## 1 Introduction

A homicide is classified as sexual when it meets at least one of the criteria established by the definition developed by the FBI. These criteria include: (a) the victim's clothing or lack thereof, (b) the exposure of the victim's sexual organs, (c) the sexual positioning of the victim's body, (d) the insertion of foreign objects into the victim's body cavities, (e) evidence of sexual intercourse, or (f) indications of substitute sexual activity, interest, or sadistic fantasy (Ressler et al. 1988). Over the years, many systems for classifying sexual homicides have been proposed to simplify the diverse nature of these offenders and assist in criminal investigations (Beauregard and Proulx 2002). Typologies offer several potential benefits. Firstly, sexual homicide typologies serve as valuable tools in investigative profiling. By collecting crime scene data and victimology information, law enforcement can develop offender profiles, aiding in suspect apprehension (Ressler et al. 1988). Secondly, if the offender is captured, these systems can inform the criminal justice system about the offender's level of violence and potential for reoffending (Prentky and Burgess 2012). Lastly, developing an offender typology can guide those involved in treatment planning and clinical practice. Therefore, it is essential to empirically investigate and validate these classification systems (Helfgott 2008). However, with so many systems in existence, where should we start?

Before analyzing the existing typologies, it is crucial to consider how sexual homicide has been conceptualized. From a strictly theoretical standpoint, two opposing perspectives can explain sexual homicide. The first perspective views sexual homicide as the result of escalating violence during a sexual assault (Mieczkowski and Beauregard 2010). According to this view, homicide and criminal violence exhibit the same behavior and processes, differing only in the outcome. In other words, the dynamics of homicide and other assaults are identical, but the former results in the victim's death (Harries 1990). This perspective suggests that there are no distinctive behavioral patterns when examining severe sexual assaults that result in either physical injury or the victim's death. It also assumes that offenders who kill their victims in a sexual context do not differ from those who commit sexual assault without committing murder. Regardless of the offender's initial intent, the actual outcome depends on the interaction between the offender and the victim (Tedeschi and Felson 1994).

The second perspective posits that sexual homicides are committed by a specific type of offender (Healey et al. 2016). According to this view, distinctive dynamics in homicides are connected to the formation of lethal intent. “From this point of view, a substantial portion of homicide offenders truly intend to kill their victims, not merely to injure them. Consequently, the victim's death is not an incidental outcome reflecting extraneous considerations but is an integral part of the incident that is likely to be systematically related to other aspects of that incident” (Felson and Messner 1996, p. 520). This perspective, therefore, suggests that significant differences in the characteristics of offenders may underlie this lethal intent.

An alternative approach to conceptualizing sexual homicide is to distinguish between homicides that are sexually motivated (i.e., the offender was motivated by sexual gratification) and those merely associated with sexual activity. Grubin (1994) offers examples of situations where a homicide is related to sexual activity but not necessarily driven by sexual motivation: eliminating a potential witness after a rape, overcoming a victim's resistance during a rape, accidently killing the victim during a rape, and participating in a rape‐homicide with accomplices.

These various conceptualizations of sexual homicide underscore the significant heterogeneity inherent in this form of extreme violence. Consequently, it is unsurprising that numerous typologies have been proposed over the years in an attempt to categorize this type of offender. However, many of these typologies have been developed in isolation, resulting in new labels for offenders who appear to share similar characteristics. Furthermore, most empirically‐based typologies have not been replicated, leading to a proliferation of classifications for this extreme form of homicide (Beauregard et al. 2007). This review aims to assess the developments of the past 25 years regarding sexual homicide typologies and to identify the key characteristics that should be included in a comprehensive and inclusive typology of these offenders.

### 1.1 Classifications, Typologies, and Taxonomies

Although often used interchangeably, classifications, typologies, and taxonomies refer to distinct concepts. According to Bailey (1994; as cited in Cale 2018), typologies are multidimensional and conceptual classification systems. They allow for the identification of multiple types based on various criteria, which are obtained through the permutation and/or combination of specific dimensions for descriptive purposes. In contrast, taxonomies are classification systems derived from quantitative methods and are based on empirically observable and measurable indicators (Bailey 1994). As Cale (2018) notes, typologies and taxonomies can be interconnected; typologies can be viewed as pseudo‐theoretical frameworks that represent initial steps in organizing phenomena according to key dimensions, while taxonomies provide the empirical evaluation of these concepts, leading to either validation or refinement of earlier ideas (Brennan 1987).

### 1.2 Organized/Disorganized Classification

In the 1970s, the FBI developed classification systems for crime scene profiling consistent with the first‐generation classifications, focusing on categorizing sexual homicides into two distinct types: organized and disorganized. This framework was derived from interviews and case studies involving 36 male sexual murderers (Ressler and Burgess 1985; Ressler et al. 1986; Ressler, Burgess, Hartman, et al. 1986). Built on the offender's crime scene behavior and background characteristics, the FBI's model remains one of the most referenced typologies in the field of sexual homicide. Despite substantial criticism regarding the methodology and validity of this typology (Canter et al. 2004; Kocsis et al. 2002), it has significantly contributed to the evolution of criminal profiling and influenced subsequent classification systems.

According to Ressler et al. (1988), an organized offender typically leads a methodical, well‐ordered life, which is mirrored in their meticulous planning and execution of a crime. They carefully prepare for the offense, often avoiding detection by removing forensic evidence, transporting the weapon to and from the crime scene, and hiding the victim's body. Such offenders are likely to use restraints, choose a victim they can control, and ensure the victim complies with their commands. Typically, organized murderers are intelligent, socially adept, and sexually competent, capable of manipulating and luring victims through charm and conversational skill. They may even impersonate authority figures to gain the victim's trust (Douglas et al. 2013; Ressler et al. 1988). Douglas et al. (2013) also note that organized offenders might take souvenirs from the crime scene and stage it to mislead investigators into thinking it was a disorganized attack.

In contrast, disorganized offenders often exhibit below‐average intelligence, social inadequacies, and sexual incompetence. Their crime scenes reflect a lack of planning, appearing chaotic and haphazard (Ressler et al. 1988). These offenders may randomly choose victims and launch surprise attacks. Disorganized crime scenes typically contain more physical evidence, such as blood or fingerprints, and the weapon is often one of opportunistic selection, left at the scene. The victim's body might be mutilated or display excessive violence, with no restraints used as the crime is usually quick and brutally executed. While post‐mortem mutilation is common among disorganized offenders, post‐mortem dismemberment can be associated with either category depending on the offender's motive. Dismemberment of the victim committed in order to dispose of the body and hide evidence is representative of the organized offender (Meloy 2000), while dismemberment resulting from violence and overkill is more typically linked to the disorganized offender.

### 1.3 The Usual Suspect: Sadistic Type

While the organized‐disorganized classification is informative and useful for profiling purposes, it has faced several criticisms. Issues such as bias in case selection and the absence of multivariate statistical techniques for identifying types (Beauregard et al. 2007) highlight the limitations associated with this classification. As a result, researchers in the field of sexual homicide have developed alternative typologies that are more consistent with the second generation of classifications. Although most of these typologies identified at least two types that resemble the organized and disorganized categories, some also recognized additional types.

One of the most consistent type reported in most typologies is the sadistic category (e.g., Ted Bundy), which aligns closely with the characteristics of the organized type. According to Beauregard (2018), in the context of the crime, sadistic sexual homicides are typically marked by offenders harboring elaborate and overpowering fantasies, which they use during the planning stages. These criminals “hunt” for their victims, carefully selecting individuals who meet certain criteria through surveillance. It is common for them to consume alcohol before committing the crime, often precipitated by a blow to their self‐esteem or a situational stressor. Sadistic sexual homicides often involve manipulation to approach an unknown victim. Offenders may use a vehicle to commit the crime and typically select an isolated crime scene, predetermined and distant from their home. Their methods reflect sadistic fantasies, including the use of torture instruments or a “rape‐kit,” tying and gagging the victim, and engaging in prolonged, ritualized torture, such as genital mutilation. Sexual acts, including fellatio, vaginal and anal penetration, may be recorded alongside the murder, often caused by strangulation. Sadistic sexual homicides can also involve unusual acts like the insertion of objects into body cavities, dismemberment, and keeping trophies or souvenirs from the victim. After the murder, offenders may choose to move the victim's body to conceal it and delay detection. They might relocate to another city or change jobs, while some may even volunteer to assist in the investigation. Some show interest in media coverage of the crime, yet their behavior remains largely normal in the aftermath. They typically express no remorse and may even derive pleasure from describing their horrific acts.

### 1.4 Beyond Sadism: Anger and Eliminating the Witness

One type of sexual homicide that has been frequently identified in previous classifications, and shares many characteristics with the disorganized type, is the angry sexual homicide (e.g., Andrew Cunanan). As summarized by Beauregard (2018), this type of homicide is characterized by an explosive and violent attack on a victim known to the offender, who is typically older than the perpetrator. The offender usually accesses the crime scene on foot, a location familiar to them and often outdoors. Restraints are minimally used, but a weapon discovered at the scene may be utilized during the crime, although the cause of death is often strangulation, similar to sadistic sexual homicides. Such homicides can be triggered by the victim's words or actions, leading to humiliation and extreme violence, including blows to the face and excessive force. There may be a sexual assault on the victim, especially post‐mortem acts, yet no semen is typically found at the scene (Beauregard et al. 2007). Angry sexual homicides are marked by a lack of planning and an impulsive urge to kill, fueled by displaced rage toward the victim. Victims are usually selected opportunistically during the offender's daily activities and in familiar locations. Offenders commonly experience suicidal thoughts, depressive moods, and anger prior to the crime (Beauregard et al. 2007). Those who commit an angry sexual homicide usually leave the victim's body lying on their back and in plain view at the crime scene. Unlike offenders of sadistic homicides, those who commit angry sexual homicides show no interest in media coverage of the crime and often report feeling relieved after the murder (Beauregard et al. 2007).

Another type of sexual homicide identified in several studies involves offenders who kill their victims primarily to eliminate witnesses, with sexual assault being their primary intent and the murder serving as a means to an end (e.g., Gerald Stano). These offenders are often noted for seldom having long‐term emotional relationships, and their victims are typically unknown to them and younger than 30. The sexual assault frequently involves penetration and may include some sadistic elements. The murder can be either impulsive or calculated, depending on the offender's level of criminal experience. Usually, the victim's wounds are limited to a single area on the body, and the victim is typically found lying on their back. The crime is often committed, and the victim's body discovered, at the initial point of contact between the offender and the victim (Beauregard et al. 2007).

### 1.5 Issues With Typologies on Sexual Homicide

While informative, most of the typologies identified have significant limitations. Firstly, many of these typologies are based on a very small sample size. This not only restricts the analyses that can be conducted but also means that small samples are usually not representative, making their findings difficult to generalize. Additionally, some studies have focused on extreme cases (such as sadists and serial murderers) or have used convenience sampling, including only offenders who agreed to participate (Beauregard and Proulx 2002). Another issue with earlier typologies concerns the variables examined. Some studies have centered on offenders, while others have investigated a limited set of behavioral characteristics in sexual homicide. Few studies incorporate a wide range of variables, making comprehensive portraits of the phenomenon rare. Moreover, some studies have combined serial and single cases, overlooking the significant differences between serial and non‐serial sexual murderers (James and Proulx 2014). Similarly, many typologies mix cases based on victim types (such as children, women, and men), despite research showing that the offending process can vary greatly depending on the type of target (e.g., Chopin and Beauregard 2020b, 2023). Chan and Heide (2009) suggest that it would be beneficial to distinguish between sexual murderers who target specific victim groups, like children or sex trade workers (see Beauregard and Martineau 2016). They further argue that meaningful conclusions about sexual murderers can only be drawn if the study stratifies the sample based on victim type.

These issues, combined with the diverse offender profiles and varying methodological approaches, underscore the fragmentation within the field and emphasize the urgent need for a more cohesive framework. The complexity of sexual homicides and the range of perspectives shaping their classification make it clear that existing systems are often disconnected. This situation highlights the necessity for a systematic approach to consolidate and synthesize the existing research. A scoping review is therefore essential for assessing sexual homicide typologies, given the significant heterogeneity of this crime and the variety of classification systems that have emerged. A scoping review would provide a comprehensive examination of the literature, identify key characteristics across typologies, and synthesize the findings. Such a review is crucial to consolidate knowledge, address methodological gaps, and support the development of more empirically grounded and inclusive typologies.

## 2 Aim of the Study

Since the publication of the organized/disorganized typology in 1986 (Ressler et al. 1988), numerous alternative typologies have been proposed, each attempting to address the limitations associated to the FBI model. Specifically, over the last 25 years, various sexual homicide typologies have been developed, each proposing different numbers of types, labels, methodologies, and characteristics. While some similarities exist among these typologies, most have been developed independently, often disregarding the contributions of others. The primary objective of this study is to review the research conducted over the past quarter‐century to determine how we can identify the most comprehensive typologies of sexual homicide. Specifically, the study explores the following research questions:

R1: How many types a sexual homicide typology typically include to adequately represent the entire phenomenon?

R2: Are there specific types that are more prevalent and should be included in a sexual homicide typology?

R3: What are the main characteristics included in a representative typology of sexual homicide, and which methods have been used to identify these types?

## 3 Methods

### 3.1 Overview

For this review on the typologies of sexual homicides, we have chosen the scoping review methodology due to its suitability for exploring complex and multi‐dimensional subjects. Unlike systematic reviews, which are designed to address narrowly defined research questions with stringent inclusion criteria, a scoping review allows for a broader and more flexible investigation of the literature (Munn et al. 2018). This approach is particularly pertinent to the study of sexual homicide typologies, given the diverse array of sources available—ranging from academic publications and governmental reports to criminal justice case studies—which highlight the varied and evolving nature of this topic.

This scoping review encompasses literature published from 2000 to 2024, concentrating on the identification, classification, and analysis of sexual homicide typologies. The year 2000 was selected as the starting point because it marks the beginning of the proliferation of empirical typologies in the field. The objective is to examine, evaluate, and synthesize research findings from both peer‐reviewed articles and gray literature that address the categorization of sexual homicides, with a particular focus on the various methodologies and frameworks employed across studies. The methodology guiding this review is based on the principles outlined in the SCIE (Systematic Research Reviews: Guidelines), which informed our search strategy, data extraction, and evaluation process (Rutter et al. 2013). Consistent with best practices for scoping reviews, we adhered to the PRISMA‐ScR (Preferred Reporting Items for Systematic Reviews and Meta‐Analyses extension for Scoping Reviews) checklist at all stages of the review process, including identification, screening, eligibility, and inclusion (Liberati et al. 2009; Moher et al. 2009).

We utilized Covidence software for our analysis, a tool specifically designed to facilitate scoping reviews and noted for its efficiency in managing large volumes of literature (Harrison et al. 2020; McKeown and Mir 2021). This approach allowed for a comprehensive and structured examination of existing research on sexual homicide typologies, offering insights into classification frameworks and their application across various jurisdictions and contexts.

### 3.2 Definition

To ensure clarity in our methodological approach, we define sexual homicide typologies as classification systems or frameworks used to categorize various forms of sexual homicides based on specific characteristics and behavioral patterns (Chan 2015). In this study, examining sexual homicide typologies involves analyzing the distinct methods, motives, and offender‐victim dynamics that differentiate various types of sexual homicides. These typologies aim to capture the diversity of sexual homicides by considering factors such as the nature of the sexual assault, the level of premeditation, the offender's profile, and the circumstances surrounding the crime.

### 3.3 Inclusion and Exclusion Criteria

Table 1 outlines the inclusion and exclusion criteria used to select studies for our review. Initially, an exploratory phase was conducted to identify relevant literature. The inclusion criteria were intentionally broad to encompass any studies related to sexual homicide typologies (Rutter et al. 2013). In contrast, exclusion criteria were established to refine the selection to studies closely aligned with our research focus. Our two‐step selection process involved first compiling studies based on the inclusion criteria, followed by the application of the exclusion criteria. While all contributions to the field are valuable, the exclusion criteria helped us pinpoint a more focused set of studies directly pertinent to our research questions on sexual homicide typologies.

**TABLE 1:** Inclusion and exclusion criteria.

Criteria	Inclusion	Exclusion
Language	English	Studies not published in English (e.g., Italian, French)
Time frame	After 2000	Studies published before the year 2000
Study focus	Studies directly related to the typologies of sexual homicides	Studies focusing on other forms of homicide or unrelated topics
Peer‐review status	Peer‐reviewed materials (e.g., books, chapters, peer‐reviewed journals, research reports, PhD theses)	Non‐peer‐reviewed materials (e.g., conference proceedings, websites, gray literature)
Relevance	Studies focusing on the classification and typologies of sexual homicides	Studies focusing on legal frameworks or typologies of non‐sexual homicide/sexual assault with non‐lethal outcome
Methodological rigor	Studies employing contemporary methodologies with robust data analysis (e.g., multivariate analysis, machine learning)	Studies with outdated or anecdotal methodologies lacking methodological transparency

Firstly, we excluded studies not published in English. Secondly, we limited our analysis to studies published after 2000, excluding earlier works due to significant advancements in theoretical frameworks relevant to the study of sexual homicides. This period marks important developments in data collection practices and standardized classification systems within criminal justice databases, improving the precision and consistency of research. Excluding older studies ensures the review focuses on literature employing contemporary methodologies, which are better suited to address the complexities of modern sexual homicides. Since 2000, significant methodological advancements have occurred in areas such as offender profiling, crime scene analysis, and psychological assessment, facilitating more nuanced and precise classifications of sexual homicides. Modern methodologies benefit from improved data availability and sophisticated statistical tools, allowing researchers to analyze larger datasets and apply advanced techniques, such as multivariate analyses and machine learning models. These innovations enhance the ability to detect patterns, identify correlations, and develop typologies that reflect the diversity and complexity of sexual homicides. Moreover, relying on typologies developed from statistical methods improve the ability of other researchers to replicate their findings. Thirdly, we concentrated on official academic materials, including books, chapters, peer‐reviewed journal articles, research reports, and PhD theses, to ensure the reliability and academic rigor of the sources. We excluded materials that had not undergone peer review (e.g., conference proceedings, websites), as these sources may not have been subject to the same level of scrutiny, raising concerns about their validity and reliability. This decision was made to maintain a high level of methodological integrity and avoid potential biases from anecdotal or non‐peer‐reviewed sources. Moreover, gray literature often lacks the methodological transparency necessary for ensuring replicability and robust comparative analysis. Fourthly, we excluded studies focusing on topics outside the scope of sexual homicides, such as those examining other forms of homicide, unrelated forensic methods, or legal discussions concerning criminal justice processes. Legal frameworks and commentaries were excluded because, while they provide important context for prosecuting sexual homicides, they were considered peripheral to the primary objective of this review: understanding and classifying the various typologies of sexual homicide. By applying these criteria, we refined our dataset to include only the most pertinent and rigorously evaluated studies, thereby enhancing the overall quality and relevance of our scoping review.

In accordance with the Systematic Research Reviews: Guidelines (SCIE; Rutter et al. 2013), exclusion criteria were applied to the studies that reached the eligibility phase (*n* = 43). The inclusion and quality criteria described below were subsequently employed to finalize the selection of studies that met all requirements (*n* = 19).

### 3.4 Search Strategy

The search strategy aimed to identify relevant academic studies from multiple databases—PubMed, Scopus, and Web of Science—with the final selection imported on May 6, 2024. The following keyword combinations were used across all databases to explore the topic of sexual homicide typologies:

- (“Sexual homicide” OR “Sexual murder”) AND (typology OR classification) AND empirical
- (“Sexual homicide” OR “Sexual murder”) AND (“empirical study” OR “empirical research”) AND (typology OR classification)
- (“Sexual homicide” OR “Sexual killing”) AND (typology OR classification) AND (data OR results)
- (“Empirical evidence” OR “quantitative” OR “qualitative”) AND (“Sexual homicide” OR “Sexual murder”) AND typology
- (“Sexual homicide” OR “Sexual murder”) AND (classification OR typology) AND (“study” OR “analysis” OR “investigation”)
- (“Sexual homicide” OR “Sexual killing”) AND (typology OR classification) AND (“case data” OR “statistical analysis”)
- (“Sexual homicide” AND “methodology”) AND (typology OR classification) AND empirical
- (“Sexual murder” AND “research”) AND (typology OR classification) AND (“empirical findings” OR “field study”)

These combinations of search terms, employing Boolean logic and truncation techniques, enabled a comprehensive exploration of the relevant literature on sexual homicide typologies. This approach allowed for a broad yet focused search, facilitating the identification of studies that empirically examine and classify sexual homicides (Rutter et al. 2013). Additional strategies included manually reviewing abstracts and conducting targeted searches in Google Scholar using the same terms to ensure no key studies were overlooked.

**FIGURE 1:**
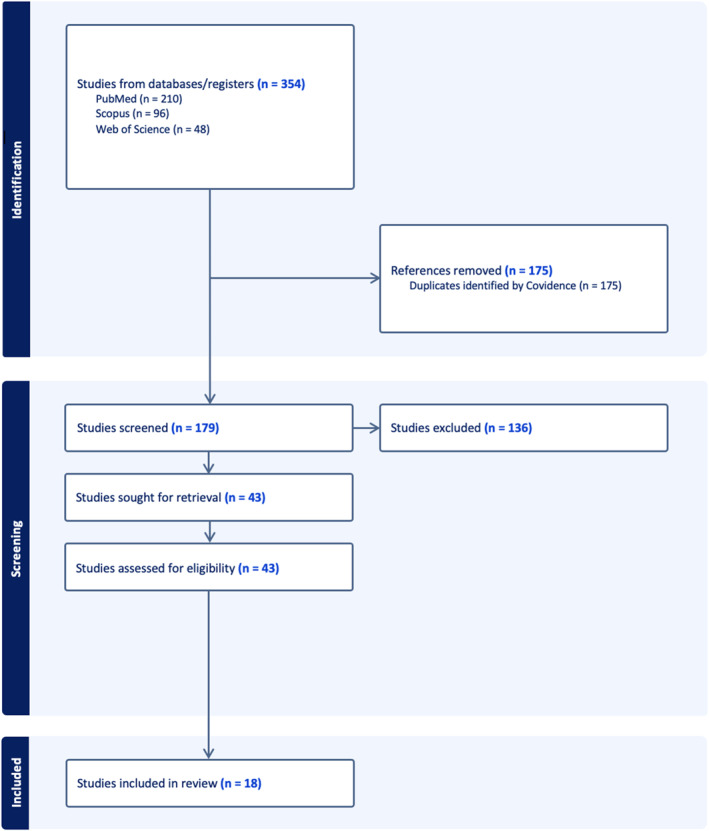
PRISMA flow diagram outlining the number of studies identified, screened and deemed (in)eligible at each stage of the review process.

All retrieved studies underwent a rigorous screening process based on the predefined inclusion and exclusion criteria (Table 1). Figure 1 presents the PRISMA flow diagram, which details the process of refining search results using these criteria.

### 3.5 Quality Appraisal

The quality of the selected studies was assessed using the SCIE guidelines (Rutter et al. 2013). Two key criteria were applied during the evaluation process. The first criterion focused on the relevance of the research to the subject of the review, ensuring that the studies were directly related to sexual homicide typologies. Studies that specifically examined the classification of sexual homicides based on offender behavior, psychological traits, or crime scene characteristics were deemed highly relevant. Conversely, studies addressing general violent crime typologies without specific reference to sexual homicides were excluded to maintain a clear focus on the research question.

The second criterion concerned the appropriateness of the research design. This involved evaluating whether the methodologies employed in each study were suitable for addressing the research question. Empirical studies using quantitative approaches, such as statistical analysis of crime data, or qualitative methods, such as interviews with offenders, were considered robust. In contrast, studies lacking rigorous methodological frameworks or those based purely on anecdotal or superficial case reports were judged less suitable. This selection criterion ensured to limit our analysis to empirical typologies that could be more easily replicated by other researchers. The review also considered whether ethical standards were followed, especially in studies involving sensitive subjects like sexual homicides, ensuring that ethical considerations such as data anonymization were in place.

The final set (*n* = 19) is composed of references that present findings from various studies. Results were systematically extracted and analyzed following the principles of narrative synthesis (Popay et al. 2006).

## 4 Results

Table 2 provides a summary of the countries, number of cases, data types, identified types, methods, labels, and key variables used in empirically derived sexual homicide typologies published between 2002 and 2024. In total, 19 typologies have been identified within this period. Of these, five originate exclusively from Canada, while eight result from collaborations between France and Canada. The remaining typologies were developed by researchers from France, South Africa, the UK, Israel, and one represents a collection of international cases. The analysis reveals that the majority of typologies were identified using police data. Of the 19 typologies, 13 relied exclusively on police data. Three additional typologies were identified through interviews with offenders, while the rest used a combination of police, court, and open‐source data (e.g., Wikipedia, encyclopedias). Unsurprisingly, only two typologies were identified through qualitative methodologies. Consequently, 17 of the 19 typologies employed statistical techniques, primarily cluster analysis (*n* = 6) or latent class analysis (*n* = 8). The remaining typologies were derived using multidimensional scaling, principal component analysis, or neural network analysis. The analysis also indicates significant variation in the number of participants across studies. As shown in Table 2, the smallest sample used to identify a typology of sexual homicide offenders included 8 participants, while the largest included 662 cases. More than half of the typologies (*n* = 10) in this analysis involved 100 or more cases. Of the 19 typologies, 11 involve a mixed variety of victims such as women, men, and children in sexual homicide cases. Only three typologies focus exclusively on women as victims. The other typologies specifically address cases involving children, men, the elderly, and juveniles.

**TABLE 2:** Summary of empirically derived classifications of sexual homicide, 2002–2024.

Authors	Year	Country	Data	N	Type of case	Methods	# Of types	Labels	Variables
Beauregard & proulx	2002	Canada	Offender interview	36	Women	K‐means cluster analysis	2	Sadistic; angry	**Victim**: Stranger; **crime**: Planning; victim selection; humiliation; mutilation; physical restraints; offense duration is more than 30 min; body left at crime scene; high risk of apprehension.
Beauregard & proulx	2007	Canada	Offender interview	10	Men	Qualitative	3	Avenger; sexual predator; nonsexual predator	**Offender**: Age; marital status; education level; occupational problems; relational problems; intoxication; pornography consumption; deviant sexual fantasies; **victim**: Stranger; age; intoxication; residential situation; sexual orientation; criminal antecedents (i.e., sexual, nonsexual violent, property), versatility; **crime**: Planning; victim selection; searching for specific characteristics; accomplice; kidnapping; weapon; physical restraints; resistance; use of physical violence; anal sex; mutilation; humiliation; high risk of apprehension; offense duration is more than 30 min; body moved or hidden;
Sewall, Krupp, & Lalumiere	2013	International	TruTV, wikipedia, encyclopedia of serial killers	82	Serial ‐ mixed	Principal component analysis, K‐means cluster; TwoStep cluster	4	Sadistic; antisocial; slasher; unknown	**Offender**: Sadism; paraphilia; calm versus distressed; school performance; level of education; CATS score; psychopathy; APD score; **crime**: Mutilation; disembowelment; depersonalization; ritualism; torture; physical restraints; order versus disarray; instrumental versus reactive.
Balemba, Beauregard, & martineau	2014	Canada	Police	350	Mixed	Latent class analysis	3/2	Solved: Sloppy/Reckless; violent/Sadistic; forensically aware; unsolved: Forensically aware; not forensically aware (Lucky)	**Crime**: Vaginal intercourse; item taken; presence of semen; mutilation; foreign object insertion; beating; physical restraints, gags, blindfolds; strangulation.
Horning, salfati, & Labuschagne	2015	South Africa	Police	33	Serial ‐ mixed	Multidimensional scaling	2	Victim as object; victim as vehicle	**Victim**: Gender; apart of a couple; alive; seeking work; prostitution; unidentified; child; **crime**: Type of approach; victim's residence; crime in public view; weapon; single wound; foreign object insertion; items taken; post‐mortem injuries; dismemberment; body in isolated location; physical restraints; strangulation/asphyxiation; penetration; covering victim's face; covering victim's body; transporting victim's body; posed victim's body; necrophilic acts; **offender**: Watched couple have sex.
Kerr & Beech	2016	UK	Offender interview	8	Women	Qualitative	4	Avenging sexual abuse; events leading to a catathymic reaction; homicidal impulse; emotional loneliness. Also 2 pathways: sadistic and anger.	**Offender**: Triggers of abuse; symbolic figure of the victim; anxiety; release of tension state; grievance; rejection; impotence; social awkwardness; retreating into own world; deviant fantasy
Reale, Beauregard, & martineau	2017	Canada	Police	350	Mixed	TwoStep cluster analysis	3	Sadistic; mixed; non‐sadistic	**Crime**: Sexually dominated victim; physical or psychological torture of victim; gratuitous violence used against victim; forced oral/anal sex on victim; use of inanimate objects on victim; sexually mutilated the victim; trophies/souvenirs taken from victim.
Chopin & Beauregard	2019	France & Canada	Police	72	Children	TwoStep cluster analysis	6	Intentional/prepubescent; inadvertent/prepubescent; intentional/preteen; inadvertent/preteen; indiscriminate/teen; intentional/teen	**Victim**: Stranger; **crime**: Type of approach; familiarity with places; residence; penetration; strangulation; forensic awareness; moving body.
Chopin & Beauregard	2020a	France & Canada	Police	56	Elderly	TwoStep cluster analysis	4	Sexual; robber; sadistic; experimental	**Crime**: Type of approach; introduction let in by victim; victim residence; penetration; items taken; moving body.
Chopin & Beauregard	2020b	France & Canada	Police	55	Juvenile	Latent class analysis	4	Explosive opportunistic; sadistic; overcontrolled anger; predator	**Victim**: Stranger; **offender**: Sadism; **crime**: Victim targeted; type of approach; offense outdoor; penetration; beating; weapon; strangulation/asphyxiation.
Greenall & wright	2020	UK	Police	81	Women	Multidimensional scaling	4	Rape; impersonal sexual assault; overkill; control.	**Crime**: Injuries inflicted; victim asphyxiated; weapon; victim stabbed/cut/slashed; victim stripped naked; penetration; Victim's genitals exposed; other sexual acts committed; victim engaged sexually; oral penetration; body left exposed; precautions taken; body concealed; body moved from crime scene; Victim's clothing ripped/torn; disabled the victim.
Chai, Beauregard, & McCuish	2021	Canada	Police	249	Mixed	Latent class analysis	3	Expressive; methodical; instrumental	**Victim**: Stranger; **crime**: Planning; victim targeted; mutilation; physical restraints; victim's body left at crime scene; high risk of apprehension
Chopin & Beauregard	2021a	France & Canada	Police	662	Mixed	Latent class analysis	3	Impulsive; sadistic; personal	**Victim**: Stranger; **offender**: Sadism; **crime**: Type of approach; victim targeted; weapon; beating; diversity of sexual acts; precautions taken.
Chopin & Beauregard	2021b	France & Canada	Police	109	Mixed	Latent class analysis	4	Opportunistic; experimental; preferential; sadistic	**Offender**: Single; sexual dysfunction; **crime**: Penetration; foreign object insertion; mutilation; strangulation/asphyxiation; physical restraints; victim targeted; items taken.
Chopin & Beauregard	2023	France & Canada	Police	285	Mixed	Latent class analysis	4	Anal/oral sex; inanimate object insertion; collector; torture/mutilation.	**Crime**: sexual domination; torture; degrading and/or humiliating behavior; gratuitous/excessive violence; anal/oral sex; inanimate object insertion; sexual mutilation; souvenirs or trophies.
Chopin & Beauregard	2023b	France & Canada	Police	100	Men	Latent class analysis	3	Robber; sadistic; pedophile	**Victim**: Child; **offender**: Sadism; **crime**: Victim targeted; penetration; items taken.
Oligny, Gauthier, menard, & James	2023	France	Police & court	120	Mixed	TwoStep cluster analysis	6	Sexual nonsadistic; sadistic; angry; sexual opportunistic; severe sadistic; psychopathic.	**Crime**: Planning; motivation for homicide (anger, non‐deviant sexuality, deviant sexuality); type of violence (instrumental/expressive).
Chopin & Beauregard	2024	France & Canada	Police	569	Mixed	Neural network analysis	2	Situational complexity; victimological complexity	**Victim:** Intoxication; homeless; involved in domestic activities; socializing; involved in sports activities; traveling; hitchhiking; involved in sex‐trade activities; **crime**: Type of body recovery location; penetration; beating; strangulation/asphyxiation; overkill; stabbing/cutting; dismemberment; shot; body moved; scene cleared/modified; semen found.
Dayan	2024	Israel	Court, media, police	107	Mixed	Latent class analysis	2	Nonmarginalized; socially marginalized	**Victim:** Stranger; commuting; **crime:** Crime scene location; motive (e.g., refusal to have sex); killing method (e.g., strangulation); additional crime (e.g., theft, robbery);

In addition to the information on sexual homicide typologies, and in alignment with the objectives of this scoping review, we adhered to the principles of narrative synthesis (Popay et al. 2006) to extract and analyze the results. This process led to the identification of three main themes. For the first theme – the number of types – the review demonstrates that the number of types within each typology varies from two to six. The most frequently observed typology consists of four types, as evidenced in seven studies (Chopin and Beauregard 2020a, 2021a, 2021b, 2023; Greenall and Wright 2020; Kerr and Beech 2016; Sewall et al. 2013). This is closely followed by typologies comprising three types, which are identified in five studies (Balemba et al. 2014; Beauregard and Proulx 2007; Chai et al. 2021; Chopin and Beauregard 2023; Reale et al. 2017). Furthermore, five typologies consist of two types each (Balemba et al. 2014; Beauregard and Proulx 2002; Chopin and Beauregard 2024; Dayan 2024; Horning et al. 2015). Notably, there are also two typologies that identify six types (Chopin and Beauregard 2019b; Oligny et al. 2023).

The second theme – typical types – suggests that when analyzing the labels used to describe the identified types, it is noteworthy that some labels appear more frequently than others. As shown in Table 2, the “sadistic” type is present in 11 of the 19 typologies (Balemba et al. 2014; Beauregard and Proulx 2002; Chopin and Beauregard 2020a, 2020b, 2021a, 2021b, 2023; Kerr and Beech 2016; Oligny et al. 2023; Reale et al. 2017; Sewall et al. 2013). Interestingly, these “sadistic” types are identified in cases of sexual homicide involving diverse victims, including women, men, the elderly, and mixed victim types. The “anger” sexual homicide type also consistently appears across different typologies of sexual homicide (Beauregard and Proulx 2002; Chopin and Beauregard 2020b; Kerr and Beech 2016; Oligny et al. 2023). Although different labels may be used, several other typologies feature at least one type similar to the “anger” category (Beauregard and Proulx 2007; Chai et al. 2021; Chopin and Beauregard 2020a, 2020b, 2021a, 2023; Greenall and Wright 2020; Sewall et al. 2013). For example, in the typology of sexual homicide against men by Beauregard and Proulx (2007), the authors identify the “avenger” type, which is clearly motivated by anger stemming from the offender's past experiences of abuse.

As to the third theme – typical characteristics – most typologies rely on crime‐related information to identify the various types. This information typically includes details such as crime planning, the type of approach, the sexual acts committed, the different acts of violence inflicted on the victim, crime‐related locations, body disposal methods, and the cause of death. As shown in Table 2, only one typology did not include any crime‐related details (Kerr and Beech 2016), focusing solely on the offender instead. Other typologies incorporated information about the offender; in fact, 10 of the 19 typologies used offender‐related data to identify the various types (Beauregard and Proulx 2007; Chopin and Beauregard 2020b, 2021a, 2021b, 2023; Horning et al. 2015; Kerr and Beech 2016; Sewall et al. 2013). The offender information varied among studies but included characteristics such as age, education level, marital status, and various psychological issues (e.g., paraphilias, psychological disorders, sexual dysfunctions). Lastly, only eight of the 19 typologies included victim‐related information to identify different types (Beauregard and Proulx 2002, 2007; Chai et al. 2021; Chopin and Beauregard 2019c, 2020b, 2021a, 2023; Dayan 2024; Horning et al. 2015). Victimology typically considers the offender‐victim relationship, age, gender, and whether the victim was intoxicated at the time of the crime.

## 5 Discussion

Since the publication of the FBI typology of sexual homicide – the organized‐disorganized model (Ressler et al. 1988) – several classifications of this particular type of homicide have emerged. Concentrating on the last 25 years, our review has uncovered 19 distinct classifications. Although many of these classifications vary in several respects, it is noteworthy to observe their similarities. More importantly, examining these 19 classifications enables us to analyze what an ideal typology of sexual homicide might resemble.

First and foremost, it is important to note that most of the typologies developed over the past quarter‐century have originated from Canada and France. The collaboration between these two countries has generated a wealth of new and innovative knowledge about sexual homicide (e.g., Chopin and Beauregard 2020a, 2021b, 2023; Chopin and Beauregard 2024). However, it is essential for other countries to also develop typologies based on their own case studies. While cultural factors appear to have limited influence on sexual homicide (Chopin and Beauregard 2019a; Sea et al. 2019; Skott et al., 2019), it is crucial to further investigate this aspect in various contexts, particularly in non‐Western countries. Nations such as South Korea (Sea et al. 2019; Sea et al. 2019) and China (e.g., Chan 2023, 2024; Chan et al. 2019) possess the necessary data to identify patterns of sexual homicide within their populations. Conducting such studies would be valuable in confirming the most common patterns of sexual homicide and determining whether cultural influences have an impact on these patterns.

Another important finding is the source of the data used in these studies. While most typologies rely primarily on police data, the analysis of existing typologies suggests the necessity for a combination of various data sources. Police data provides valuable information about the crime itself (e.g., modus operandi, context) and the victims, but it often lacks comprehensive details about the offenders (Chopin and Beauregard 2019c). In contrast, the research conducted by Beauregard and Proulx (2002) demonstrated the benefits of accessing both types of data—police information and offender interviews. Not only does interview data help confirm certain facts related to the cases, but it also captures specific details about the offenders, particularly regarding their personalities. Therefore, integrating both data sources could lead to a more complete and nuanced typology of sexual homicide. Similarly, previous classification studies on sexual homicide have mainly employed cluster analysis and latent class analysis to identify the different types of crimes. While both methods are robust and possess distinct advantages (Collins and Lanza 2009, 2010; Lanza et al. 2007; Lanza et al. 2003), other methodologies have also demonstrated their utility. Recent research on sexual homicide has highlighted the significant complexity surrounding these offenses and the necessity of considering the various interactions among the included variables. Therefore, future classification studies should explore the application of machine learning techniques (e.g., regression trees, neural network analysis) more thoroughly (e.g., Chopin and Beauregard 2024).

### 5.1 Two Is Not Enough

Focusing specifically on our first research question, the most frequently identified number of types in sexual homicide typologies is either three or four. This contrasts with the organized‐disorganized model proposed by the FBI (Ressler et al. 1988). However, even the FBI recognized additional types—or subtypes—related to their dichotomous model. For example, Ressler et al. (1988) suggested that the organized category includes a subtype of sadistic sexual homicide. Furthermore, in response to various criticisms of the dichotomous model, the FBI introduced the “mixed” type to their typology to account for cases that do not fit neatly into either of the two original categories (Beauregard and Proulx 2002). This suggests that the FBI's initial identification of two main types remains relevant today. However, subsequent typologies have managed to identify a greater number of types, indicating that these primary categories may actually encompass subtypes that provide more nuanced characterizations of these cases. For instance, in a study by Chopin et al. (2022), the authors found that even sadistic offenders could be further classified into distinct subcategories. A comprehensive classification system should explore whether the identified types include both main categories and their corresponding subtypes.

### 5.2 Sadistic as a Constant

Related to our first research question, our second research inquiry was aimed at determining whether certain types of sexual homicide are more prevalent than others. This question is more challenging to address, as many typologies intentionally use labels that differ from those in previous studies. However, one type that consistently appears across most typologies is the sadistic type. In 11 out of the 19 typologies examined, researchers identified a sadistic category (Balemba et al. 2014; Beauregard and Proulx 2002; Chopin and Beauregard 2020a, 2020b, 2021a, 2021b, 2023; Kerr and Beech 2016; Oligny et al. 2023; Reale et al. 2017; Sewall et al. 2013). Furthermore, the characteristics used to define this type were remarkably similar across different studies, leading the authors of these typologies to retain the same label. This is significant, as it suggests that the sadistic type represents a major category within sexual homicide, even though, as previously noted, it may encompass subtypes as well (Chopin et al. 2022). The sadistic type has frequently been associated with the organized category identified by the FBI (Beauregard and Proulx 2002). Likewise, the “angry” type has been identified in several typologies, which may share numerous characteristics with the disorganized type (Beauregard and Proulx 2002; Chopin and Beauregard 2020b; Kerr and Beech 2016; Oligny et al. 2023). Similar to the sadistic type, it is plausible that other identified types also represent subcategories of the angry sexual homicide.

### 5.3 Offense and Victim Characteristics

Regarding our third research question, it is evident that nearly all typologies, with the exception of one, were based on information pertaining to the offense itself. This indicates that a comprehensive typology of sexual homicide should incorporate at least some variables that describe the nature of the crime committed. In terms of offender and victim characteristics, the situation is less clear. Likely due to the reliance on police data in most of the typologies, only 8 out of the 19 classifications included details about the offender. This prompts the question of whether offender characteristics should be an essential component of the typology or merely used as variables to assess the external validity of the typology. Additionally, it raises the consideration of whether we should focus on typologies of sexual homicides or typologies of sexual murderers.

When it comes to victim characteristics, it's important to highlight that among the 19 typologies, 11 include a diverse range of victims, encompassing women, men, and children in sexual homicide cases. Only three typologies concentrate exclusively on women as victims. Having various types of victims within the same typology could potentially cloud the findings, especially given that numerous studies have identified significant differences in sexual homicides involving women compared to those involving children and men (e.g., Chopin and Beauregard 2022, 2023). As Chan and Heide (2009) argue, meaningful conclusions about sexual murderers can only be drawn if the study stratifies the sample based on victim type. Nevertheless, even if a robust typology focuses solely on one type of victim, previous studies suggest that there are advantages to considering other victim characteristics as well. For example, research has demonstrated that certain victim‐related characteristics can provide additional insights into the context of the crime and the associated level of risk (Ressler et al. 1988).

The analysis of the 19 existing typologies of sexual homicide allows us to summarize the key victim and crime characteristics that have been used to identify the primary types of sexual homicide. Beyond the victim's gender and age, these typologies have taken into account factors such as the victim‐offender relationship, sexual orientation, whether the victim was part of a couple, and the victim's residential status, such as homelessness. Additional victim‐related information includes intoxication levels, criminal history (e.g., sexual offenses, nonsexual violent crimes, property crimes), and routine activities (e.g., prostitution, domestic activities, socializing, recreational activities, hitchhiking, commuting).

Information regarding the crime itself can be categorized into three main stages. During the pre‐crime stage, typologies of sexual homicide examine elements such as crime planning, victim selection, whether the victim was specifically targeted, and any specific characteristics sought in a victim. Additionally, these typologies consider the presence of accomplices and the motivation behind the homicide, whether it stems from anger, non‐deviant sexuality, or deviant sexuality.

The crime stage is where the majority of crime‐related details emerge. Typologies typically encompass the sexual acts committed, such as oral, vaginal, anal, or other sexual acts. They also investigate the type of violence used (e.g., instrumental or expressive), the approach method (e.g., con, blitz, surprise), and instances of extreme violence (e.g., physical or psychological torture, mutilation, disembowelment, dismemberment, insertion of foreign objects, overkill). Most typologies also include information on actions taken by the offender to commit the crime, such as beating the victim, using physical restraints, gags or blindfolds, kidnapping, employing a weapon, covering the victim's face and/or body, strangulation, stabbing/cutting, and either partially or fully undressing the victim (e.g., exposing victim's genitals). Other crime phase characteristics include the presence of humiliation, the duration of the offense, victim resistance, depersonalization, ritualism, as well as locations associated with the crime (e.g., victim's residence, public vs. private spaces).

In the post‐crime phase, most typologies consider body disposal methods (e.g., body left at the crime scene, body moved or hidden), risk of apprehension, the presence of semen, and whether or not the offender took items belonging to the victim (e.g., trophies or souvenirs). Furthermore, some typologies include information about post‐mortem injuries, necrophilic acts, posing the victim's body, and precautions taken to evade police detection (i.e., forensic awareness).

## 6 Conclusion

Although sexual homicide is a highly specific type of homicide, a significant number of typologies have been proposed over the years. In the past 25 years alone, 19 empirical typologies on this topic have been published. This is noteworthy given the specificity of the offense. However, due to the combination of the rapid emergence of new typologies and the isolated research efforts, these classification systems were unable to incorporate all the new ideas that emerged. Although new findings were presented, they were often not integrated into the subsequent typological work. This has resulted in typologies with varying labels and differing content. Furthermore, methodological choices made in these studies have not always aligned with the existing understanding of the heterogeneity in sexual homicide. This scoping review was essential to consolidate the accumulated knowledge on sexual homicide typologies over the past quarter‐century. We believe the review can play a crucial role in guiding future efforts to propose a new and more comprehensive typology of sexual homicide, especially now that we have access to larger samples and more sophisticated statistical methods for analyzing data on these cases.

Despite our best efforts, we acknowledge that this review has limitations. By restricting the scoping review to English literature, we may have excluded important typologies published elsewhere. Similarly, typologies developed in other, especially non‐Western, countries may not have been published in scientific journals, resulting in a lack of diversity in the typologies we analyzed.

Since the FBI introduced the organized‐disorganized typology, substantial progress has been made in understanding this type of homicide. Typologies are a valuable tool for enhancing our understanding of a specific phenomenon. We hope that this work will guide future efforts toward identifying a new typology that can aid the various criminal justice practitioners involved in these cases.

## Author Contributions

**E.B.** and **J.C.** contributed to the conception and design of the study, data collection, and drafting of the manuscript. **E.B.** conducted data analysis and interpretation. **J.C.** assisted in the design of the study and provided significant input in drafting and finalizing the manuscript. **E.B.** contributed to the acquisition of funding and overall supervision of the project.

## Ethics Statement

The study was conducted in accordance with the relevant guidelines and regulations at Simon Fraser University. Ethical approval was not required for this study because the data analyzed consisted of publicly available research articles.

## Conflicts of Interest

The authors declare no conflicts of interest.

## Data Availability

Author elects to not share data. Due to the sensitive nature and the origin of the data, it is not possible for the researchers to share the research data.
